# Rerouting Eye Care: How AI and Telemedicine Are Reshaping Ophthalmology Patient Journeys in the United Kingdom and Germany

**DOI:** 10.2196/93170

**Published:** 2026-05-27

**Authors:** Broder Poschkamp, Ariel Yuhan Ong, Justin Engelmann, Michael Grün, Andreas Stahl, Pearse Andrew Keane, David Adrian Merle

**Affiliations:** 1 Department of Ophthalmology, Universitätsmedizin Greifswald, Ferdinand Sauerbruch Street, Greifswald, Mecklenburg-Vorpommern, 17475, Germany, 49 3834865900; 2 Institute of Ophthalmology, University College London, London, England, United Kingdom; 3 Moorfields Eye Hospital NHS Foundation Trust, London, England, United Kingdom; 4 Oxford Eye Hospital, Oxford University Hospitals NHS Trust, Oxford, England, United Kingdom; 5 NIHR Moorfields Biomedical Research Centre, London, England, United Kingdom; 6 Department of Ophthalmology, St. Franziskus Hospital, Münster, North Rhine-Westphalia, Germany; 7 Center of Ophthalmology, University Hospital Tuebingen, Tuebingen, Germany

**Keywords:** patient pathways, artificial intelligence as a medical device, tele-ophthalmology, ophthalmic artificial intelligence, ophthalmic AI, artificial intelligence, AI screening

## Abstract

Artificial intelligence (AI) as a medical device is now progressively entering routine ophthalmic care, yet its impact is still mostly evaluated in terms of diagnostic performance rather than how it reshapes patient care pathways. This viewpoint argues that careful pathway design is crucial to implementing AI in ophthalmology so that it translates into real, practical benefits for patients and services. We propose a framework that classifies AI- and telemedicine-enabled eye care pathways by the initial point of contact and the role of AI: from direct human assessment to grader- or telemedicine-based use to AI-first contact and fully autonomous AI gatekeeping. We apply this framework to 2 contrasting health systems, the United Kingdom and Germany, focusing on common retinal diseases and cardiovascular risk assessment from retinal images. In the United Kingdom, AI is being introduced mainly as a modular upgrade to standardized programs layered onto a gatekeeper model centered on community optometrists and general practitioners. In Germany, direct access to office-based ophthalmologists, opportunistic screening, and commercial retail offerings are producing more fragmented clinician- and market-driven AI adoption. Our comparison shows that the same AI technologies can generate very different patient journeys across health systems. Therefore, their value depends not only on diagnostic performance but also on intentional pathway design, including clear escalation rules and structured information transfer. This includes appropriate task shifting from ophthalmologists to trained staff or new roles, such as image grading, tele-ophthalmology triage, or pathway management. Without such design, AI risks duplicate testing, incomplete or poorly coordinated referrals, and further fragmentation of care.

## Introduction

Artificial intelligence (AI) has emerged as a powerful tool for ophthalmic screening since around 2018, following the first pivotal trials of autonomous diabetic retinopathy (DR) systems [1]. It is now available in multiple regulatory jurisdictions across the European Union, the United Kingdom, China, India, and other regions [2,3]. Current regulatory approvals for artificial intelligence as a medical device (AIaMD) encompass the detection and monitoring of DR, glaucoma, and age-related macular degeneration (AMD), as well as emerging oculomics tasks such as biological age estimation and cardiovascular risk prediction [2]. However, despite strong diagnostic performance in controlled studies (typically retrospective), real-world uptake remains slow and heterogeneous, with substantial variation in how AI is integrated—if at all—into regional and national eye care pathways and health systems.

At the same time, the demand for eye care is rising: ophthalmology is now the busiest outpatient specialty in the United Kingdom, contributing the largest number of outpatient visits and with demand projected to rise by more than 40% over the next 20 years [4]. In Germany, over the past 15 years, demand for ophthalmic care has risen by approximately 15% to 34%, whereas effective care capacity has remained almost unchanged [5].

The technical backbone of most ophthalmic AI screening programs is digital fundus photography, which permits the detection of multiple retinal and optic nerve diseases from a single, noninvasive imaging modality [6]. The wider availability of affordable cameras and stable internet connection has enabled tele-ophthalmology models in which images captured in primary care, community clinics, or retail settings are analyzed remotely by AIaMD and/or human graders. These models support digital-first pathways that triage which patients truly require in-person specialist care [7].

In this viewpoint and conceptual analysis, we examine how the interplay between currently available AIaMD and telemedicine may reshape patient journeys for DR, AMD, glaucoma, and cardiovascular risk prediction in the United Kingdom and Germany. We selected these 2 high-income health systems because they have similar burdens of these conditions but fundamentally different access structures. The UK health care system is comparatively more centralized and nationally coordinated, whereas the German system is more fragmented. Both countries are early adopters of AIaMD and tele-ophthalmology, yet they differ substantially in their care structures. This contrast makes them well suited to illustrate how the same technologies can generate divergent patient journeys. We first introduce a pragmatic framework to classify ophthalmic care pathways by care setting and level of AI autonomy. We then apply this framework to current and potential pathways in both health systems. Finally, we outline clinical, organizational, and policy implications for designing future AI- and telemedicine-enabled eye care pathways that streamline patient access to eye care while maintaining safety and equity. This viewpoint is intended for clinicians, service designers, policymakers, and other stakeholders involved in implementing AI-enabled eye care pathways.

## Conceptual Framework

In this viewpoint, “screening” denotes first-line examinations in asymptomatic at-risk individuals to detect previously undiagnosed eye disease. “Surveillance” refers to repeated examinations to monitor patients with known or suspected disease or high-risk groups over time to trigger treatment or retreatment [8]. “Tele-ophthalmology” describes remote eye care using digital imaging and/or video. Within this domain, we distinguish between remote image grading, which is limited to diagnostic assessment, and telemedicine (or teleconsultation), which includes treatment capabilities such as issuing prescriptions or initiating therapy. Current AIaMD can be applied to autonomous diagnostic tasks such as image grading and to the surveillance of existing disease over time.

In this paper, a care pathway refers to the overall sequence from first presentation to diagnosis, referral, treatment, and follow-up, whereas “triage” denotes one step within that pathway that determines prioritization or onward referral. For our comparison of UK and German patient journeys, we introduce the term “retail-based screening” to describe eye assessments performed in supermarkets, optometry shops, or pharmacies outside traditional medical practices.

**Figure 1.**
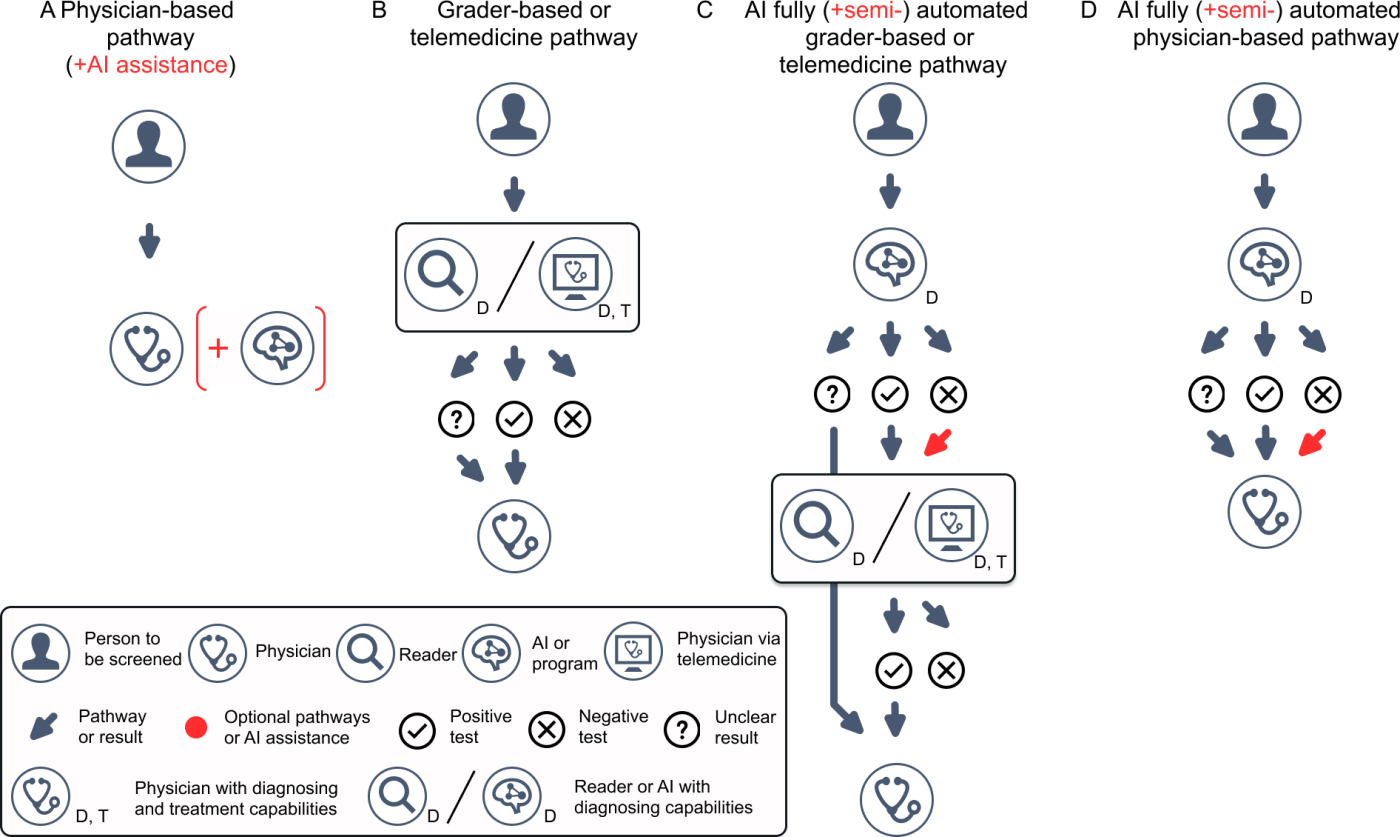
Four generic artificial intelligence (AI)–supported care pathways are distinguished. Pathway A is a physician-based pathway in which the patient is seen in person by an ophthalmologist; AI may be used in the background, but the patient does not have direct AI contact. In pathway B, the first contact is digital, for example, via image grading by a grader or a telemedicine consultation with a physician. Pathway C is a confirmatory model in which AI provides the initial assessment, followed by review by a grader or telemedicine physician before any in-person consultation. Pathway D is a model in which an AI system first analyzes all patients and, in a fully automated manner, decides which patients need to be seen by a physician. In pathways C and D, red arrows indicate whether a human review step is present (semiautomated) or absent (fully automated), with absence indicating a higher level of autonomy.

We distinguish 4 generic AI-enabled screening pathways (Figure 1). Whether AI only assists a grader or independently decides who is referred for further treatment determines whether a pathway is classified as semiautomated or fully autonomous. Pathway A represents conventional care, where the patient is examined directly by an ophthalmologist; AI may be used by the physician in the background, but it is not visible in the direct patient contact. In pathway B, images or cases are first assessed by a grader or through a telemedicine contact, which then decides whether referral to an ophthalmologist is required. In pathway C, an AI provides an initial assessment, but cases are still reviewed by a grader or via telemedicine before patients reach the treating ophthalmologist (semiautomated model: every case is seen by a human, in contrast to fully automated pathways where only a subset of cases is forwarded). In pathway D, AI makes the primary screening decision without a grader and decides which cases are referred for in-person treatment.

## Patient Journeys in the United Kingdom

The United Kingdom’s National Health Service (NHS) is a tax-funded national health system that provides universal coverage and generally free care at the point of use, with community eye care delivered largely through general ophthalmic services contracts and hospital eye services funded directly from NHS budgets. The NHS operates on a strict “gatekeeper” model, where access to specialist ophthalmology care is controlled by primary care providers rather than permitting direct specialist access. UK patients generally cannot consult a hospital ophthalmologist without a formal referral except in cases of acute emergency. While direct access to ophthalmologists is possible via the private practice route, this is not available in the public sector.

**Figure 2.**
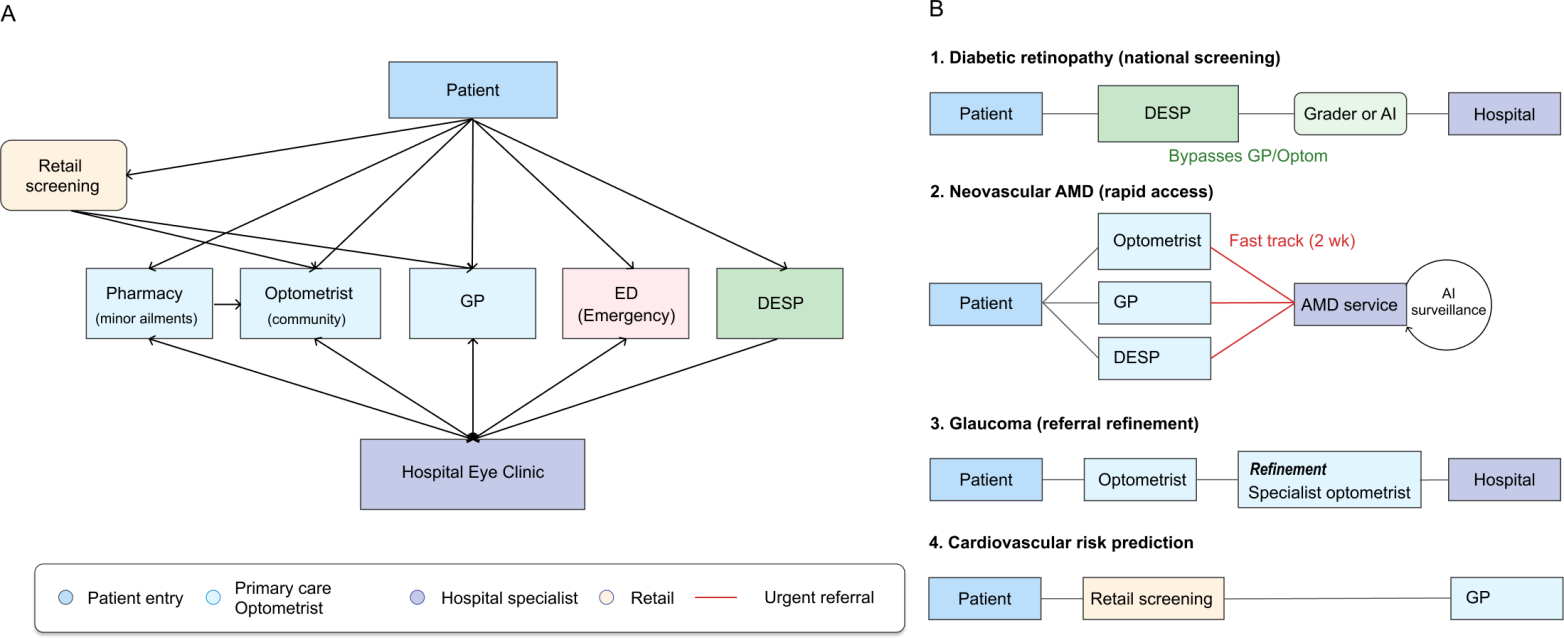
Patient journeys in the United Kingdom. (A) The United Kingdom follows a gatekeeper model, meaning that patients usually cannot access hospital eye clinics directly. Patients with eye problems enter via pharmacy minor ailment schemes (often with referral to community optometry), general practitioners (GPs), or emergency departments (EDs), which can refer onward to the hospital eye clinic. Patients with diabetes may also be referred via the diabetic eye screening program (DESP). (B) Artificial intelligence (AI) as a medical device–enabled pathways are disease and service specific. In diabetic retinopathy, image assessment in the DESP (by a grader and/or AI) can trigger referral to hospital, bypassing the GP or optometry. In suspected neovascular age-related macular degeneration (AMD), referrals can originate from optometry, GPs, or the DESP and are channeled via an urgent fast track (approximately 2 weeks) to an AMD service. The glaucoma pathway may include refinement by specialist optometrists before hospital escalation, and retail screening can also direct patients into National Health Service care (eg, via the GP). Red arrows indicate urgent referral routes.

This structural constraint has necessitated specific detection and referral pathways to manage the flow of patients into secondary care. In this model, the community optometrist plays a significantly expanded clinical role compared to those in many other European nations, acting as the primary triage filter for the health system [9]. As the vast majority of these optometrists operate within commercial retail environments (“high-street optometry practices”), the UK model represents a public-private interface where retail technology adoption directly influences medical pathways. Patients presenting with visual symptoms or those attending for routine refraction generally visit a community optometrist first. If pathology is suspected, the optometrist issues a referral to the hospital eye service. In the context of our framework, this standard UK model represents conventional care (pathway A) but with a unique community-based prescreening layer performed by optometrists rather than physicians (Figure 2A).

For DR, the patient journey shifts significantly from this opportunistic model to a systematic, active screening approach. The United Kingdom supports one of the world’s most mature tele-ophthalmology programs, the NHS diabetic eye screening program (DESP), with each of the 4 nations (England, Scotland, Wales, and Northern Ireland) operating its own DESP with slightly different modes of operation [10]. All eligible individuals with diabetes are invited to dedicated community clinics or mobile units for regular screening. In this workflow, digital fundus images are captured by technicians and transmitted to a centralized grading center, where they are reviewed by trained human graders following a strict national protocol. This represents a telemedicine or human grading (pathway B) model. The English DESP is currently validating fully autonomous grader-based filtering models (pathway C), in which AI systems act as a filter to exclude “no disease” images (Figure 2B) [11], whereas the Scottish DESP has already deployed such an automated first-line grading system (eg, iGradingM) since 2011 (pathway C) [12].

In contrast, the journey for patients with neovascular age-related macular degeneration (nAMD) is usually symptom driven and time critical. Patients typically present to a primary care optometrist with acute symptoms such as distortion or central vision loss. To minimize delay, optometrists use “fast-track” electronic referral pathways to hospital eye services to achieve the national target of a specialist assessment within 2 weeks of referral [13]. These referrals can come from general practitioners (GPs) or from the DESP as well [14]. This represents a streamlined conventional care approach (pathway A); unlike the DR pathway, there is no intermediate telemedical grading step for diagnosis as the clinical suspicion raised by the optometrist triggers an immediate face-to-face specialist assessment. Recent data from a randomized clinical trial showed that embedding a tele-ophthalmology step into this urgent macula referral pathway can substantially reduce false-positive urgent referrals [15]. In parallel, an AI-enabled decision tool for treatment monitoring in nAMD is currently being prospectively evaluated within NHS retina clinics explicitly focusing on clinical safety and pathway integration (Figure 2B) [16].

Glaucoma detection in the United Kingdom remains largely opportunistic, occurring primarily during routine sight tests at optical shops, many of which are NHS funded for eligible risk groups such as individuals with a first-degree relative with glaucoma [17]. Historically, this approach has generated high false-positive referral rates due to the difficulty of diagnosing glaucoma based on intraocular pressure or optic disc appearance alone. To mitigate this, many regions commission glaucoma referral refinement services [18]. In this workflow, a patient flagged by a standard optometrist is not referred directly to the hospital but is instead re-examined by a specialist community optometrist with advanced qualifications. Only if this specialist confirms the suspicion is the patient referred to secondary care. This constitutes a sophisticated variation of conventional care (pathway A), introducing a community-based human filter to triage cases (Figure 2B) [19].

The United Kingdom is beginning to introduce oculomics into the eye care pathway to identify systemic health risks, although these applications remain exploratory, with limited evidence to date [20]. In this emerging model, AI algorithms analyze retinal fundus images captured during routine retail eye examinations to predict cardiovascular risk or risk factors [21,22]. Early deployments have been reported mainly in private optometry practices and private clinics, where cardiovascular risk scores are offered as an add-on to standard eye examinations on a self-pay basis outside formal NHS commissioning [23]. In the context of our framework, this corresponds to a fully autonomous digital-first pathway (pathway D), in which an AI system generates a systemic risk estimate that could, in principle, prompt direct referral to a GP for cardiovascular assessment and management as necessary. However, these services remain at an exploratory stage, and uptake in routine optometric practice is limited, with outstanding questions regarding reimbursement models, workflow integration, governance, and professional responsibility (Figure 2B) [20].

## Patient Journeys in Germany

**Figure 3.**
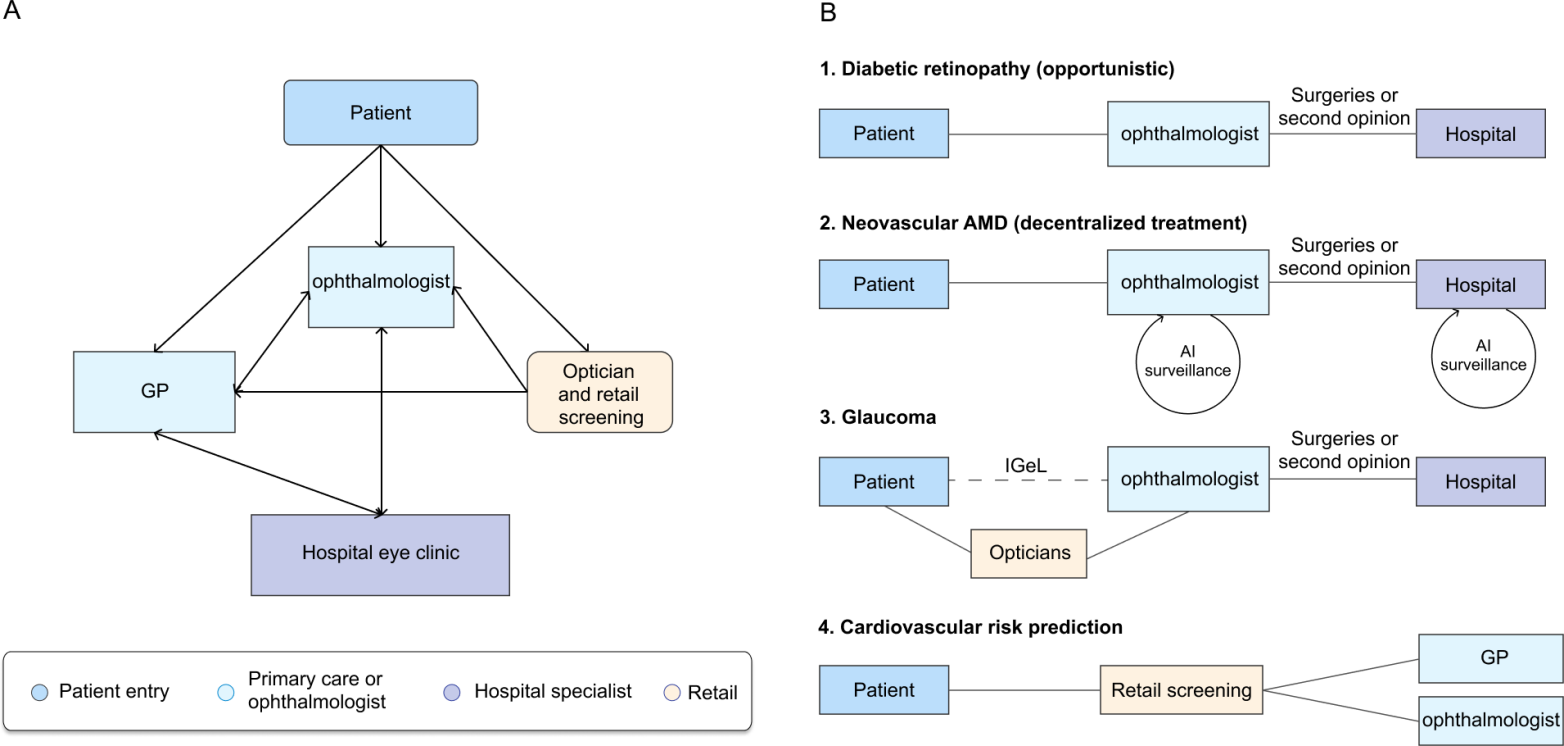
Patient journeys in Germany. (A) In Germany, patients can directly consult an ophthalmologist. Entry may also occur via a general practitioner (GP) or via opticians or retail screening, which can direct patients to ophthalmology. Hospital eye clinics are typically involved for surgery and second opinions rather than first contact. (B) Artificial intelligence (AI) as a medical device–enabled pathways are disease and service specific. Diabetic retinopathy is detected during opportunistic examinations by ophthalmologists, with escalation to hospital when required. In neovascular age-related macular degeneration (AMD), care can remain decentralized, with possible AI-supported surveillance. Glaucoma includes additional entry routes (eg, opticians and self-paid or individual health services [IGeL]) before specialist assessment, with hospital involvement mainly for surgical care or second opinions. For cardiovascular risk prediction from retinal images, retail screening can direct patients onward to a GP or ophthalmology. *Surgeries or second opinion.

In Germany, health care is mainly financed through statutory health insurance (GKV) with income-related contributions and near-universal coverage, with outpatient eye care provided largely by office-based ophthalmologists reimbursed by sickness funds and hospital eye services funded via statutory insurance and diagnosis-related group–based hospital payments [24]. In contrast to the United Kingdom’s gatekeeper model, the German health care system is defined by a direct-access specialist structure. Patients seeking eye care possess the freedom to consult an office-based ophthalmologist directly [25]. However, the provision framework is more complex than a purely physician-based model. While eye care services are formally provided by ophthalmologists (and, to a minor extent, by GPs), opticians play a nonnegligible role in the initial contact. Although legally classified within the handicraft sector rather than as health care professionals, German opticians are entitled to perform screening tests, including tonometry, perimetry, and examinations of the anterior and posterior segment provided they refer any suspected abnormalities to a physician for formal diagnosis [26]. While the specialized role of the optometrist is gaining importance, it is not yet as widely available or as clearly defined in scope as in the United Kingdom. Consequently, despite this informal prescreening layer, the final human filter in Germany remains the ophthalmologist. In the context of our framework, the standard German model corresponds almost exclusively to conventional care (pathway A), where the final screening decision, diagnosis, and management are performed face-to-face by a physician (Figure 3A) [27].

For DR, Germany relies on an opportunistic screening model. While national guidelines mandate annual or biannual eye examinations for patients with diabetes with and without comorbidities (eg, arterial hypertension or diabetes known for >10 years), respectively, the responsibility lies with the patient and their diabetologist to ensure that these visits occur [28]. Screening is performed exclusively by office-based ophthalmologists using slit lamp biomicroscopy with or without fundus photography (pathway A). Consequently, telemedicine or AI integration is almost nonexistent in routine care and remains largely limited to specific pilot projects [29]. More recently, retail-based screening models have introduced an additional semiautomated (all cases are reviewed), grader-based pathway (pathway C) [30]. For DR, hospital eye care is only necessary for surgery or complex cases or to provide a second opinion (Figure 3B).

The journey for nAMD in Germany is similarly shaped by office-based specialists. Patients noticing visual changes typically self-refer directly to their local ophthalmologist. Many German office-based ophthalmologists are equipped to administer intravitreal injections themselves or work in close networks with specialized injection centers [27]. Ophthalmologists in both office-based practices and hospital clinics can equip their imaging devices with AI tools that assist with AMD biomarker segmentation and classification [31]. However, these are pilot implementations, and they represent a physician-based pathway with AI support that does not meaningfully change the patient journey (Figure 3B).

Glaucoma detection follows an opportunistic and often commercially driven logic. As GKV does not routinely cover asymptomatic glaucoma screening, intraocular pressure measurements and optic disc assessments are frequently offered as individual health services, which patients must pay for out of pocket [24]. This creates a socioeconomic filter rather than a clinical one. When a patient accepts this screening, the diagnosis is confirmed directly by the ophthalmologist. Unlike the United Kingdom’s referral refinement schemes, which use specialist optometrists to filter false positives, the German system absorbs this diagnostic load directly within the primary specialist layer (pathway A; Figure 3B).

The use of oculomics for cardiovascular disease risk assessment is only beginning to enter the German market and, at present, is confined to exploratory offerings in the retail optical sector [32]. As GKV does not yet provide reimbursement for retinal cardiovascular screening, this innovation currently follows a commercial pathway corresponding to pathway D. Similar to the United Kingdom, patients pay out of pocket at an optician for a retinal scan that can provide both an AI analysis of DR, glaucoma, and AMD and an AI-based cardiovascular risk assessment [33]. While such models illustrate how high-technology oculomics screening could be brought into community settings, they currently operate without formal integration into GKV-funded care pathways or clearly defined referral arrangements to GPs, cardiologists, or ophthalmologists (Figure 3B).

## Digital-First Pathways

Digital-first models, particularly those emerging in the retail sector, create new entry points into eye care largely operated by nonmedical staff. While these services can improve access, they must not function as diagnostic dead ends without clear pathways to treatment or clinical decision-making. For DR, programs in which screening results are directly linked to a scheduled ophthalmology appointment have been shown to achieve substantially higher attendance rates (up to 5-fold higher) compared with models that only ask patients to make an appointment with an eye care specialist [34].

Critically, patients with acute “red flag” symptoms, such as sudden vision loss, new flashes and floaters, or severe ocular pain, should bypass routine screening workflows and be directed immediately to urgent specialist assessment rather than being delayed in retail or digital screening pathways [35]. This also implies that screening services themselves should not routinely route patients into the emergency department. In the United Kingdom, tele-ophthalmology services that triage urgent referrals have demonstrated the ability to reduce the burden on hospital eye departments by safely triaging cases to reduce hospital referrals from the community [15].

AI tools used in digital-first pathways should explicitly state their intended use, for example, whether they are designed for fully autonomous screening, semiautomated decision support, or purely as a grading aid. This intended use influences acceptable thresholds for sensitivity and specificity, legal responsibility, and how results are communicated to patients [36]. In addition, those implementing and governing AI-enabled services must define the intended downstream action of AI outputs. In the emerging field of oculomics, where retinal images are used to estimate systemic risk such as cardiovascular disease, it is particularly important to clarify and define the management pathway, for example, whether a high-risk result should trigger referral to a GP, cardiologist, or ophthalmologist rather than simply labeling patients as “high risk” without a clear pathway for follow-up.

## Task Allocation and Information Transfer

For digital-first models to improve care rather than fragment it, examinations that trigger onward referral must provide sufficient structured information for the next clinician in the chain. AI reports should include key clinical variables (eg, relevant systemic risk factors, image quality, suspected diagnosis and severity, urgency, and recommended follow-up interval) rather than only a binary “refer/no refer” flag [14,28]. This is analogous to structured referral forms used in German diabetes eye care pathways, which record systemic risk factors such as diabetes type and duration, hemoglobin A_1c_ level, blood pressure, vascular complications, and an overall risk stratification, or to structured AMD referral templates in UK guidance. These reports should also be designed for seamless integration into existing electronic health record systems to ensure continuity and accessibility of information. Explainability may also be useful in this regard. Clinicians receiving or triaging the referral may wish to access the original image and the AI outputs to build trust in the AI system’s decisions.

## Comparison Between the United Kingdom and Germany

Although the United Kingdom and Germany are both early adopters of ophthalmic AIaMD and tele-ophthalmology, the organizational context in which these technologies are deployed differs substantially. In the United Kingdom, digital and tele-ophthalmology services are increasingly layered onto a gatekeeper-based system in which community optometrists and GPs act as primary entry points into eye care and control access to hospital eye services [37]. These differences influence where AI is inserted, who retains oversight, and whether implementation occurs as part of coordinated pathways or as isolated diagnostic add-ons.

In Germany, in contrast, many patients can access office-based ophthalmologists directly [24], and there is currently no significant nationwide reimbursement framework for AI-based medical devices in ophthalmology. In addition, most AI use is confined to pilot projects or local physician-based decision support tools that do not fundamentally alter the patient journey [38]. Given Germany’s direct access to specialist care, physician-based AI (pathway A) may help save clinical time, but without coordinated pathway design, its use remains fragmented and largely dependent on individual ophthalmologists’ willingness and capacity to integrate these tools [39]. Table 1 summarizes the key structural contrasts between the 2 systems and their implications for AI-enabled ophthalmology pathways.

**Table 1.** Artificial intelligence (AI)–enabled ophthalmology pathways in the United Kingdom and Germany.

Dimension	United Kingdom	Germany
Health system organization	More centralized; nationally coordinated	More fragmented; regionally variable
Entry into eye care	Gatekeeper based	Direct specialist access
Main first contact	Community optometrist, GP^a^, or organized screening	Office-based ophthalmologist
Existing tele-ophthalmology infrastructure	Stronger, especially in DR^b^ screening	Limited; mainly pilot based
Current AI deployment pattern	More at the pathway level and programmatic	More local; physician or market driven
Position of human oversight	Often before specialist referral	Often at specialist level, who is the first contact
Likely AI implementation by use case	DR: AI-supported screening or grader prefiltering within organized programs; AMD^c^: referral refinement or treatment monitoring support; glaucoma: community-based referral refinement and risk stratification; oculomics: retail- or optometry-based cardiovascular risk estimation with unclear downstream integration	DR: opportunistic practice-based screening support or retail prescreening; AMD: physician-facing imaging decision support in specialist care; glaucoma: self-pay screening or in-practice risk support; oculomics: retail or self-pay cardiovascular risk assessment with unclear downstream integration
Main risk	Complex escalation and accountability across tiers	Fragmentation and lack of pathway integration; high number of false positives
Main policy priority	Safe scaling within existing referral systems	Building coordinated pathways around otherwise isolated AI tools

a GP: general practitioner.

b DR: diabetic retinopathy.

c AMD: age-related macular degeneration.

At the same time, in both the United Kingdom and Germany, emerging retail-based digital-first services are beginning to create retail-initiated pathways (pathways C and D), typically as self-pay services that have huge potential to reduce ophthalmologist workload. In both countries, many imaging tests (eg, color fundus photography and optical coherence tomography) are reimbursed only for specific indications and otherwise paid out of pocket. If retail screening is not integrated into shared care pathways and data flows, these risks duplicate testing and double charging.

## Conclusions

Our comparison shows that the same AI technologies can lead to very different care pathways across health care systems. Therefore, to deploy AI at scale and translate it into meaningful benefit for patients and services, the next steps are not only better algorithms but also intentional pathway design: defining where AI sits in the journey, who receives its outputs, how follow-up is organized, how use is reimbursed, and how responsibility and oversight are shared. In practical terms, this includes establishing processes for low-quality or ungradable images, integrating AI outputs into routine records, and specifying clear escalation routes and follow-up intervals for urgent or uncertain findings. Experience from other countries and health system contexts, including India and broader implementation efforts across Europe, Australia, and America, suggests that ophthalmic AI can improve access and support screening but only when workflow design, referral pathways, and local health system context are addressed alongside algorithm performance [2,3,7]. Implementation may also require new roles in grading, tele-ophthalmology triage, referral refinement, and pathway management, together with the safe and feasible transfer of selected routine tasks from highly specialized ophthalmologists to appropriately trained staff. Future work should focus on co-designing and prospectively evaluating AI-enabled pathways with patients, clinicians, and payers in each system so that digital-first models improve access and efficiency without compromising safety or professional accountability.

## Abbreviations

AI: artificial intelligence
AIaMD: artificial intelligence as a medical device
AMD: age-related macular degeneration
DESP: diabetic eye screening program
DR: diabetic retinopathy
GKV: German statutory health insurance
GP: general practitioner
nAMD: neovascular age-related macular degeneration
NHS: National Health Service
